# 200. Antimicrobial Activity of Ceftazidime-Avibactam and Comparators against AmpC Hyperproducing *Enterobacterales* and *P. aeruginosa* Collected from United States (US) Medical Centers (2016-2020)

**DOI:** 10.1093/ofid/ofab466.200

**Published:** 2021-12-04

**Authors:** Helio S Sader, Cecilia G Carvalhaes, Rodrigo E Mendes, Mariana Castanheira, Mariana Castanheira

**Affiliations:** 1 JMI Laboratories, North Liberty, Iowa; 2 JMI Laboratories, Inc., North Liberty, Iowa

## Abstract

**Background:**

*E. cloacae* species complex (ECL)*, S. marcescens* (SM), *C. freundii* species complex (CF), and *P. aeruginosa* (PSA) are common pathogens in a variety of clinical infections. These organisms can overexpress the chromosomal AmpC that encodes resistance to several β-lactams. We evaluated the activity of ceftazidime-avibactam (CAZ-AVI) and comparators against these organisms.

**Methods:**

17,650 isolates, including 4,400 ECL, 2,074 SM, 1,644 CF, and 9,532 PSA, were consecutively collected from 88 US medical centers in 2016-2020. Among these isolates, 3,127 were ceftazidime-nonsusceptible (CAZ-NS; MIC ≥8 mg/L for *Enterobacterales* [ENT] and ≥16 mg/L for PSA) and considered probable AmpC hyperproducers. Isolates were susceptibility tested by broth microdilution method.

**Results:**

Susceptibility to CAZ ranged from 73.6% (ECL) to 97.5% (SM; Table). Overall, 99.8% of ENT (99.7-99.9%) and 97.1% of PSA were CAZ-AVI-S; whereas 84.3% (79.0-97.7%) of ENT and 97.4% of PSA were ceftolozane-tazobactam (C-T)-S, 83.0% (78.5-94.8%) of ENT and 80.0% of PSA were piperacillin-tazobactam (PIP-TAZ)-S, and 98.4% (98.3-98.7%) of ENT and 79.5% of PSA were meropenem (MEM)-S. CAZ-AVI retained potent activity and broad spectrum against CAZ-NS ENT (n=1,629; MIC_50/90_, 0.5/1 mg/L; 99.0%S overall) and CAZ-AVI was more active than MEM (MIC_50/90_, 0.06/0.5 mg/L; 93.1%S) against these organisms. C-T (MIC_50/90_, 8/ >16 mg/L; 23.8%S) and PIP-TAZ (MIC_50/90_, 64/ >64 mg/L; 21.8%S) exhibited limited activity against CAZ-NS ENT. Among comparator agents, only amikacin (99.0%S), tigecycline (95.6%S), and imipenem (92.1%S) showed good activity against CAZ-NS ENT. Also, CAZ-AVI retained activity against 86.7% of ENT isolates that were NS to CAZ and MEM (n=113). CAZ-AVI (MIC_50/90_, 2/4 mg/L; 97.1%S) and C-T (MIC_50/90_, 0.5/2 mg/L; 97.4%S) were the most active compounds tested against PSA and both retained activity against CAZ-NS PSA. CAZ-AVI (MIC_50/90_, 4/16 mg/L; 81.8%S) and C-T (MIC_50/90_, 2/8 mg/L; 83.9%S) activity against CAZ-NS PSA was comparable to tobramycin (MIC_50/90_, 1/ >8 mg/L; 82.2%S).

**Conclusion:**

CAZ-AVI demonstrated potent activity and broad spectrum against AmpC hyperproducer organisms, such as ECL, SM, CF, and PSA, from US hospitals and remained highly active against CAZ-NS isolates.

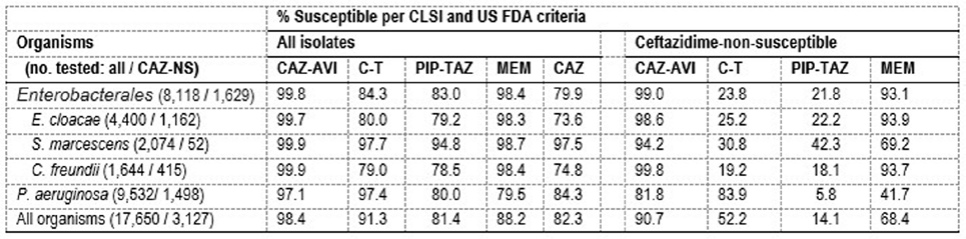

**Disclosures:**

**Helio S. Sader, MD, PhD, FIDSA**, **AbbVie (formerly Allergan**) (Research Grant or Support)**Basilea Pharmaceutica International, Ltd.** (Research Grant or Support)**Cipla Therapeutics** (Research Grant or Support)**Cipla USA Inc.** (Research Grant or Support)**Department of Health and Human Services** (Research Grant or Support, Contract no. HHSO100201600002C)**Melinta Therapeutics, LLC** (Research Grant or Support)**Nabriva Therapeutics** (Research Grant or Support)**Pfizer, Inc.** (Research Grant or Support)**Shionogi** (Research Grant or Support)**Spero Therapeutics** (Research Grant or Support) **Cecilia G. Carvalhaes, MD, PhD**, **AbbVie (formerly Allergan**) (Research Grant or Support)**Cidara Therapeutics, Inc.** (Research Grant or Support)**Cipla Therapeutics** (Research Grant or Support)**Cipla USA Inc.** (Research Grant or Support)**Melinta Therapeutics, LLC** (Research Grant or Support)**Pfizer, Inc.** (Research Grant or Support) **Rodrigo E. Mendes, PhD**, **AbbVie** (Research Grant or Support)**AbbVie (formerly Allergan**) (Research Grant or Support)**Cipla Therapeutics** (Research Grant or Support)**Cipla USA Inc.** (Research Grant or Support)**ContraFect Corporation** (Research Grant or Support)**GlaxoSmithKline, LLC** (Research Grant or Support)**Melinta Therapeutics, Inc.** (Research Grant or Support)**Melinta Therapeutics, LLC** (Research Grant or Support)**Nabriva Therapeutics** (Research Grant or Support)**Pfizer, Inc.** (Research Grant or Support)**Shionogi** (Research Grant or Support)**Spero Therapeutics** (Research Grant or Support) **Mariana Castanheira, PhD**, **AbbVie (formerly Allergan**) (Research Grant or Support)**Bravos Biosciences** (Research Grant or Support)**Cidara Therapeutics, Inc.** (Research Grant or Support)**Cipla Therapeutics** (Research Grant or Support)**Cipla USA Inc.** (Research Grant or Support)**GlaxoSmithKline** (Research Grant or Support)**Melinta Therapeutics, Inc.** (Research Grant or Support)**Melinta Therapeutics, LLC** (Research Grant or Support)**Pfizer, Inc.** (Research Grant or Support)**Qpex Biopharma** (Research Grant or Support)**Shionogi** (Research Grant or Support)**Spero Therapeutics** (Research Grant or Support) **Mariana Castanheira, PhD**, Affinity Biosensors (Individual(s) Involved: Self): Research Grant or Support; Allergan (Individual(s) Involved: Self): Research Grant or Support; Amicrobe, Inc (Individual(s) Involved: Self): Research Grant or Support; Amplyx Pharma (Individual(s) Involved: Self): Research Grant or Support; Artugen Therapeutics USA, Inc. (Individual(s) Involved: Self): Research Grant or Support; Astellas (Individual(s) Involved: Self): Research Grant or Support; Basilea (Individual(s) Involved: Self): Research Grant or Support; Beth Israel Deaconess Medical Center (Individual(s) Involved: Self): Research Grant or Support; BIDMC (Individual(s) Involved: Self): Research Grant or Support; bioMerieux Inc. (Individual(s) Involved: Self): Research Grant or Support; BioVersys Ag (Individual(s) Involved: Self): Research Grant or Support; Bugworks (Individual(s) Involved: Self): Research Grant or Support; Cidara (Individual(s) Involved: Self): Research Grant or Support; Cipla (Individual(s) Involved: Self): Research Grant or Support; Contrafect (Individual(s) Involved: Self): Research Grant or Support; Cormedix (Individual(s) Involved: Self): Research Grant or Support; Crestone, Inc. (Individual(s) Involved: Self): Research Grant or Support; Curza (Individual(s) Involved: Self): Research Grant or Support; CXC7 (Individual(s) Involved: Self): Research Grant or Support; Entasis (Individual(s) Involved: Self): Research Grant or Support; Fedora Pharmaceutical (Individual(s) Involved: Self): Research Grant or Support; Fimbrion Therapeutics (Individual(s) Involved: Self): Research Grant or Support; Fox Chase (Individual(s) Involved: Self): Research Grant or Support; GlaxoSmithKline (Individual(s) Involved: Self): Research Grant or Support; Guardian Therapeutics (Individual(s) Involved: Self): Research Grant or Support; Hardy Diagnostics (Individual(s) Involved: Self): Research Grant or Support; IHMA (Individual(s) Involved: Self): Research Grant or Support; Janssen Research & Development (Individual(s) Involved: Self): Research Grant or Support; Johnson & Johnson (Individual(s) Involved: Self): Research Grant or Support; Kaleido Biosceinces (Individual(s) Involved: Self): Research Grant or Support; KBP Biosciences (Individual(s) Involved: Self): Research Grant or Support; Luminex (Individual(s) Involved: Self): Research Grant or Support; Matrivax (Individual(s) Involved: Self): Research Grant or Support; Mayo Clinic (Individual(s) Involved: Self): Research Grant or Support; Medpace (Individual(s) Involved: Self): Research Grant or Support; Meiji Seika Pharma Co., Ltd. (Individual(s) Involved: Self): Research Grant or Support; Melinta (Individual(s) Involved: Self): Research Grant or Support; Menarini (Individual(s) Involved: Self): Research Grant or Support; Merck (Individual(s) Involved: Self): Research Grant or Support; Meridian Bioscience Inc. (Individual(s) Involved: Self): Research Grant or Support; Micromyx (Individual(s) Involved: Self): Research Grant or Support; MicuRx (Individual(s) Involved: Self): Research Grant or Support; N8 Medical (Individual(s) Involved: Self): Research Grant or Support; Nabriva (Individual(s) Involved: Self): Research Grant or Support; National Institutes of Health (Individual(s) Involved: Self): Research Grant or Support; National University of Singapore (Individual(s) Involved: Self): Research Grant or Support; North Bristol NHS Trust (Individual(s) Involved: Self): Research Grant or Support; Novome Biotechnologies (Individual(s) Involved: Self): Research Grant or Support; Paratek (Individual(s) Involved: Self): Research Grant or Support; Pfizer (Individual(s) Involved: Self): Research Grant or Support; Prokaryotics Inc. (Individual(s) Involved: Self): Research Grant or Support; QPEX Biopharma (Individual(s) Involved: Self): Research Grant or Support; Rhode Island Hospital (Individual(s) Involved: Self): Research Grant or Support; RIHML (Individual(s) Involved: Self): Research Grant or Support; Roche (Individual(s) Involved: Self): Research Grant or Support; Roivant (Individual(s) Involved: Self): Research Grant or Support; Salvat (Individual(s) Involved: Self): Research Grant or Support; Scynexis (Individual(s) Involved: Self): Research Grant or Support; SeLux Diagnostics (Individual(s) Involved: Self): Research Grant or Support; Shionogi (Individual(s) Involved: Self): Research Grant or Support; Specific Diagnostics (Individual(s) Involved: Self): Research Grant or Support; Spero (Individual(s) Involved: Self): Research Grant or Support; SuperTrans Medical LT (Individual(s) Involved: Self): Research Grant or Support; T2 Biosystems (Individual(s) Involved: Self): Research Grant or Support; The University of Queensland (Individual(s) Involved: Self): Research Grant or Support; Thermo Fisher Scientific (Individual(s) Involved: Self): Research Grant or Support; Tufts Medical Center (Individual(s) Involved: Self): Research Grant or Support; Universite de Sherbrooke (Individual(s) Involved: Self): Research Grant or Support; University of Iowa (Individual(s) Involved: Self): Research Grant or Support; University of Iowa Hospitals and Clinics (Individual(s) Involved: Self): Research Grant or Support; University of Wisconsin (Individual(s) Involved: Self): Research Grant or Support; UNT System College of Pharmacy (Individual(s) Involved: Self): Research Grant or Support; URMC (Individual(s) Involved: Self): Research Grant or Support; UT Southwestern (Individual(s) Involved: Self): Research Grant or Support; VenatoRx (Individual(s) Involved: Self): Research Grant or Support; Viosera Therapeutics (Individual(s) Involved: Self): Research Grant or Support; Wayne State University (Individual(s) Involved: Self): Research Grant or Support

